# Total healthcare costs of deinstitutionalized long-term care provision in the Netherlands: an instrumental variable analysis

**DOI:** 10.1186/s12913-025-12693-x

**Published:** 2025-04-10

**Authors:** Erik M. E. Wackers, Florien M. Kruse, Hubert J. J. M. Berden, Simone A. van Dulmen, Niek W. Stadhouders, Patrick P. T. Jeurissen

**Affiliations:** 1 IQ Health, Radboud University Medical Center, Radboud Institute for Health Sciences, Kapittelweg 54, Nijmegen, 6525 EP the Netherlands; 2 Ministry of Health, Welfare, and Sport, The Hague, the Netherlands

**Keywords:** Long-term care, Healthcare costs, Nursing homes, Health policy

## Abstract

**Background:**

The Netherlands has reformed its long-term care (LTC) system to improve its financial sustainability by reducing access to in-kind nursing-home care and stimulating care within the home setting. This reform led to the rise of small-scale homelike nursing homes (SHNH) that are publicly financed by home-care packages. Small-scale care may require additional financial resources and personnel. Our aim is to compare the total healthcare costs of institutional in-kind nursing homes and deinstitutionalized home-like care homes.

**Methods:**

We conducted an instrumental variable (IV) analysis to adjust for selection effects between both groups, using nationwide data including all LTC users. We use distance to the nearest provider as an instrument. In addition, we conducted semi-structured interviews with managers and policymakers to provide context for the quantitative data and validate our findings.

**Results:**

Baseline statistics demonstrate that while LTC costs are lower in deinstitutionalized settings, total healthcare costs are higher, mainly due to additional medical costs outside the LTC system. After adjustment for observable and unobservable user characteristics, no significant cost differences were found.

**Conclusions:**

This study may suggest that deinstitutionalization of LTC provision through publicly funded SNHNs primarily resulted in selection effects and spillover effects to other healthcare sectors, while for the marginal client, no clear difference in healthcare costs was observed.

**Supplementary Information:**

The online version contains supplementary material available at 10.1186/s12913-025-12693-x.

## Background

Many countries have expanded formal long-term care (LTC) provision to accommodate ageing populations. For example, Germany introduced LTC insurance in 1995 and Japan in 2000. Expansion is generally followed by rapid cost increases. Aging populations are projected to drive LTC expenditures up to 2.7% of the gross domestic product (GDP) by 2060 from 1.5% in 2013 in the European Union [1], which puts pressure on the financial sustainability of LTC systems [2]. A country ahead of the cost curve is the Netherlands, which introduced LTC insurance in 1967 and has the highest LTC expenditures as percentage of the gross domestic product (GDP) among OECD countries (4.4% in 2021). Dutch LTC expenditures are expected to increase further in the future [3], sparking concerns about the fiscal sustainability of public LTC provision.

The Netherlands is internationally known to have a generous public LTC system that is more institutionalized than other OECD countries [4, 5]. The LTC system covers care provision, housing costs and social services for older persons, disabled and institutionalized mental care. Access to LTC is needs-tested in the Netherlands [6]. The Care Assessment Centre (CIZ) determines the care need in terms of severity on a scale from one to ten. Case severity gradually increases, although the classifications are heterogeneous. For example, classification five has a focus on dementia (psychogeriatric) care and classification six on somatic geriatric care. Tariffs for institutional nursing-home care are set and indexed annually by the Dutch Health care Authority (NZa) and include housing costs. Tariffs generally increase but not in a linear fashion (See Supplementary material 1) [7]. Options to self-purchase care through public funding have been available since 1995 via a personal budget (PB), but were expanded by the introduction of a home-care package (HCP), covering all services except housing costs, and modular care package (MCP), which covers a selection of services, excluding housing costs [8]. HCPs are typically 70–80% of the needs-tested costs of institutionalized care, as housing arrangements and additional services are expected to be privately financed [7]. In some individual cases, users are eligible for various assistance programs, e.g. transport services and house adjustments, which are provided by municipalities [8]. The MCP allows for tailoring a limited set of services at lower costs [6]. A high monthly co-payment is required for in-kind nursing-home care, with a maximum of €2.506 in 2022, and a lower monthly co-payment for publicly-funded home care (i.e. PB, MCP, and HCP), with a maximum of €913 in 2022 [7]. Co-payments are means-tested based on household income and personal wealth of the care recipient. More affluent users are generally more inclined to opt for publicly funded home care, as the difference in maximum co-payments can be used for more personalised private housing arrangements. Housing arrangements are paid out-of-pocket, whereas care services are covered by public funding.

**Fig. 1:**
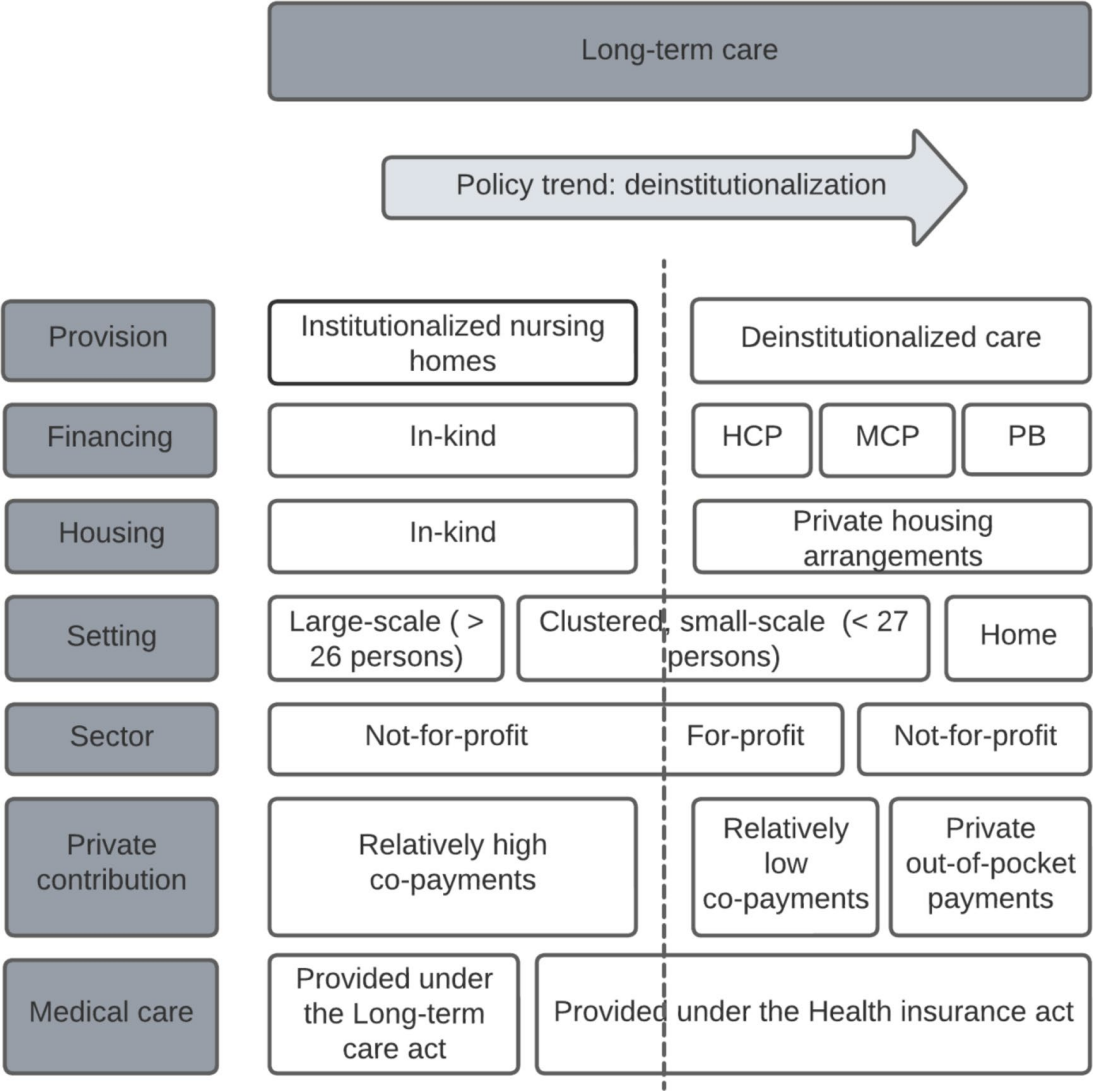
Overview of the long-term care (LTC) system in the Netherlands (HCP = home-care package; MCP = modular care package; PB = personal budget). For-profit providers are dominant in the SHNH market, whereas institutional care providers (i.e. in-kind financed nursing homes) are almost exclusively under not-for-profit ownership [6]. A high monthly co-payment is required for in-kind nursing-home care, with a maximum of €2.506 in 2022, and a lower monthly co-payment for publicly-funded home care, with a maximum of €913 in 2022 [7]

The Dutch LTC system was reformed in 2015, aiming to contain rising costs and to facilitate independent living and reduce reliance on nursing homes. Since the reform, nursing homes have been restricted to users needing 24-hour care availability, and patients with moderate needs lost eligibility for institutionalized LTC. This supported a trend towards deinstitutionalization. For this study, we define deinstitutionalization as a process by which care in traditional (institutional) nursing homes is substituted with nursing care at home and in small-scale homelike nursing homes SHNHs [9]. This study focuses on nursing care provided in SHNHs. See Fig. 1 for a brief overview of the Dutch LTC system and the deinstitutionalization trend. We refer to the paper on Dutch long-term care by Maarse and Jeurissen for an in-depth overview of the long-term care reform [8].

Institutionalized LTC provision has a strong historical base in the Netherlands, and remains the most frequently chosen option [6]. However, the reform resulted in the rise SHNHs, providing home-care within a clustered setting [6]. SHNHs consist of between 3 and 26 clients [10]. They are financed either through a PB, MCP, or HCP, although most SHNHs rely on HCP funding. For-profit providers are dominant in the SHNH market, whereas institutional care providers (i.e. in-kind financed nursing homes) are almost exclusively under not-for-profit ownership [6].

Various health and social care systems have seen trends towards deinstitutionalization, aiming to generate savings as well as more patient-centred care [4, 9, 11–13]. Policymakers assume that costs of care in home(-like) settings are lower than the costs of institutionalized care [9, 13]. However, several factors may offset the cost-containment potential. First, home care may be more accessible, increasing LTC utilization. Second, care in a home setting may increase the usage of medical care services. There is a higher chance of injury within a home setting (for example, falling leading to hip fractures), and home-care users rely more on the medical- are sector [14]. Whereas (large) nursing homes employ a geriatrician to provide medical care and the costs of this are part of the in-kind package; home-care users must turn to general practitioner (GPs) for their primary care. GPs have warned that because medical-care support in SHNHs is not sufficiently organized, the increasing number of older persons in SHNHs is increasing the burden on GPs [15]. Taking all these factors into account, deinstitutionalization likely leads to cost-shifting from the long-term care sector to the medical care sector. Thirdly, publicly funded home-care providers may cherry-pick relatively low-utilization LTC users, leading to a concentration of LTC users with higher care needs and higher costs in institutional nursing homes. This was underlined by existing qualitative and descriptive research which suggested that SHNHs have targeted a more affluent clientele [6, 16]. Empirical analysis of differences in the costs of provision could provide insight into the cost-containment effects of LTC deinstitutionalization. Our research question is: *How do SHNHs for older persons compare to institutional nursing homes with respect to total healthcare costs?*

Other studies have looked at the cost-saving potential of homecare versus institutionalized care [17–20]. While most studies have compared home-care provision to institutionalized nursing homes, to the best of our knowledge, this is the first study comparing the costs of SHNHs and institutional nursing homes while correcting for selection effects.

## Methods

### Study design

We follow a mixed methods approach. For the quantitative analyses, we analyse LTC users and assess whether user characteristics differ between SHNHs and institutional nursing homes. Nursing homes were defined as entities that provide 24-hour institutional care and support. SHNHs were operationalized as providers that cluster recipients with home-care packages and offer housing arrangements, which are privately financed. This classification allowed us to assess the effects of deinstitutionalization of nursing homes on total healthcare costs. An instrumental variable (IV) approach was used to analyse the relationship between SHNHs and total healthcare costs, controlling for potential selection bias. For the qualitative analysis, we conducted semi-structured interviews with managers and policymakers to enrich our understanding of the relationship between deinstitutionalization and healthcare costs. It can provide nuance and context to the quantitative analysis and insight in underlying mechanisms, which in turn can support policymaking.

### Quantitative data collection

We used data from 2015 to 2018 from Statistics Netherlands (CBS), covering the entire Dutch population. Individuals were pseudonymized by Statistics Netherlands using a unique identification code. We selected all individuals using publicly funded LTC for elderly (case severity levels 4–8; see Supplementary material 1). Personal characteristics include age and sex, as well as socioeconomic factors, such as personal income. Care-related characteristics include case severity, co-payments for LTC, and healthcare costs. To increase comparability, we selected deinstitutionalized care users with a HCP, excluding MCP and PB. The healthcare costs consist of both LTC costs as well as costs for primary care, hospital care, and pharmaceuticals as part of the health insurance act (HIA). Matching on (pseudonymised) address details allowed us to map address changes from private addresses to nursing home locations. As providers may offer institutional and deinstitutionalized care at the same location, an address location could be attributed to both groups. Individual users were attributed to either group based on their financing mode. Their first registered financing mode was used to categorize users to either group. Users attributed to SHNHs may likely switch to institutional nursing homes over time. This allows for analysis of total healthcare costs incurred during their length of stay.

### Outcome measures

The main outcome measures of this study were LTC costs and total healthcare costs including medical costs under the HIA using claims data. LTC costs were obtained by combining utilization data with national maximum tariffs. While purchasers may apply provider-specific discounts, limited differentiation is made between providers [21]. Housing costs were subtracted from in-kind institutional tariffs to isolate healthcare sector effects. Healthcare costs are analysed in the year after moving to a nursing home, to rule out the effect of costs as a result of adverse events prior to nursing home admission. Medical care costs are based on claims data, which include all costs under the HIA (predominantly hospital and primary care costs). In institutionalized settings, a larger share of medical care is covered under the LTC act (Fig. 1). For example, institutionalized LTC generally includes access to a geriatric specialist, while clients in SHNHs are reliant on hospital specialist services. We also analysed the length of stay (days) of users in both institutional and SHNHs. Length of stay provides an indication of healthcare utilisation and can provide insights on the mechanisms behind the drivers of total healthcare costs.

### Regression analyses

Ordinary least squares (OLS) and instrumental variables (IV) or two-stage least squares regressions were conducted. OLS regressions were used to assess the association between SHNHs and total healthcare costs corrected for observable characteristics. These results were compared to IV-analysis, which corrects for unobservable characteristics.

Selection effects may result in bias, as individuals are not randomly assigned to either an institutional nursing home or SHNH. For example, personal preference, socioeconomic status, and case severity may affect the decision to opt for either institutional care of an SHNH. An IV-analysis aims to mitigate this bias, as it mimics randomization into treatment [22]. In this study, we used differential distance as an instrumental variable. Distance to providers has been demonstrated as an important predictor for provider choice [22–24]. A study on nursing home performance in the Netherlands demonstrated that over 60% of clients opted for a nursing home within 5 km of their prior home and 21% of clients opted for the nearest location [17]. We measured the difference in distance between the private address and the nearest nursing home (either institutional or SHNH) and the distance between the private address and the nearest SHNH. We calculated the straight-line distance between addresses using coordinates. Although travel distance might be more accurate, the difference between both measures was previously demonstrated to be negligible [25]. As the differential distance approaches zero, the likelihood of choosing an SHNH increases. Differential distance was previously found an effective instrument to control for user selection in nursing homes [23, 26–28].

IV-analysis is characterized through two assumptions: (1) unobservable characteristics (self-selection) are not correlated with differential distance (random assignment), and; (2) individuals would choose another type of institution if it had been closer (monotonicity) [23]. We argue that differential distance is independent of unobservable differences that affect healthcare costs, e.g., users with high expected costs and low expected costs are equally likely to live near an SHNH. In that case, the actual observed costs of these users are only affected by the type of provision. This does require a strong correlation between distance and actual choice of the nearest provider. A study in the Dutch context did not find evidence supporting selection bias for nursing home outcomes (i.e. users opting for specific nursing home locations based on outcomes) [26]. Furthermore, earlier explorative research showed that SHNHs are more likely to be established in urban regions with a higher socioeconomic status, although regional variation is modest in the Netherlands [29]. The monotonicity assumption was tested using linear regression (Supplementary material 3, Table A3.2). We conducted the IV-analysis using two-stage least squares, where we test the effect of differential distance on nearest-provider choice in the first stage. Corrections are incorporated for observable characteristics, i.e., age, sex, income, case severity, and length of stay. Controlling for income allows for some mitigation of regional provider selection effects, i.e. SHNHs are inclined to locate in higher socioeconomic regions. We used weak instruments test (F-statistic) and Wu-Hausman postestimation tests to test the validity of our instruments [30]. The weak instruments test demonstrates whether correlation with the endogenous explanatory variable is sufficiently high (rule of thumb is F-statistic > 10). Wu-Hausman tests the consistency of an IV estimate compared to ordinary least squares (OLS) regression. Postestimation tests for all models demonstrate an F-statistic that is sufficiently high to rule out finite-sample bias [30]. Wu-Hausman test demonstrates that an OLS analysis may be more efficient than an IV analysis (Tables 2 and 3).

### Sensitivity analyses

The regression analyses were checked for robustness through exclusion of confounding variables. We also analysed models without case severity, income, and capital to assess the impact of these individual independent variables on the outcome variables. Furthermore, a subset excluding persons with more than one registered nursing home address (i.e., moved more than once to a nursing home) was used for analysis. These individuals may distort the effect on healthcare costs, as costs are calculated starting from the first entry in a nursing home. The definition of clusters of at least five individuals receiving LTC allowed us to minimize inclusion of clusters that were caused through registration errors (i.e., individuals who remain registered at the same address while they moved to another address). Higher minimal thresholds for clusters of users (e.g., ten or fifteen individuals) could result in an underestimation of locations. As sensitivity checks, these identifying conditions for SHNHs were relaxed or restricted.

### Qualitative analyses

We conducted 20 semi-structured interviews with owners, managers and representatives of SHNHs (*n* = 8), managers and representatives of traditional nursing homes (*n* = 7), healthcare insurers and policymakers (*n* = 5) between September and December 2021. We used purposive sampling to select interviewees. See Supplementary material 2 for an overview of the topic guide. These interviews were transcribed ad verbatim and subsequently analysed in ATLAS.ti (version 8.4.20). Interviews were coded thematically and based on consensus of two researchers.

## Results

### Population selection

We identified 68,186 unique persons who moved to a clustered facility (both institutional and SHNH) in the period 2016–2018. These persons had common elderly care indications (level 4–8). For reference, approximately a total of 260,000 individuals were LTC users in the Netherlands in 2019 (total population of approximately 17 million). Of these 260,000 LTC users, 13,000 users had an HCP (5%). Our estimates are that 3,5% of our identified population lived in SHNHs. Our selection excludes LTC users receiving home care (i.e. not in an SHNH) [31].

### Descriptive statistics

Table 1 shows that 96.5% of the selected users received institutional care. In terms of case severity, we note that a larger share of users with psychogeriatric indications (predominantly dementia care) is included in the deinstitutionalized group, whereas somatic geriatric indications (predominantly intensive nursing and care) make up a larger share of the institutional group. Furthermore, in line with our expectations, we find that users in SHNHs have a higher socioeconomic status. Their length of stay is also slightly longer than users of institutional settings (approximately 43 days after two years).

**Table 1:** Descriptive characteristics for institutional and deinstitutionalized homes in 2016-2018 (users who moved to a clustered facility in 2016-2018)

	Institutional	SHNHs
*Users (%)*	96.5	3.5
*Sex (%)*		
* Male*	33	29
* Female*	67	71
*Age (years*,* mean (SD))*	84.37	85.52
*Personal gross income (2018) (€*,* median)*	17,261	20,471
*Private contribution LTC (2018) (€*,* median)*	5602.56	2121.54
*Case severity (%)*		
* 4 (intensive supervision and treatment)*	12.82	14.59
* 5 (predominantly dementia care)*	56.44	69.96
* 6 (intensive care and nursing)*	24.16	12.88
* 7 (intensive care for specific conditions*,* focus on supervision)*	5.03	2.15
* 8 (intensive care for specific conditions*,* focus on care and nursing)*	1.55	0.43
*Length of stay in median days*		
* Length of stay year after admission (days)*	310.46	324.03
* Length of stay two years after admission (days)*	537.48	570.58
*Healthcare costs (€*,* median)*		
* Total medical care costs year after admission*	128.60	3137.72
* Total LTC costs year after admission*	59034.10	58827.05

*SHNH* Small-scale homelike nursing home

LTC costs make up for most of the total costs in both groups. Users in SHNHs have relatively higher medical care costs and lower long-term care costs in the year after admission.

### Association between financing type, healthcare costs and length of stay

We used OLS and IV regressions to estimate the association between SHNHs or institutional LTC provision and healthcare costs (Table 2). OLS regression demonstrates a negative association between SHNHs and LTC costs in the year after admission. These results are in line with baseline statistics. However, after correcting for selection effects using IV analysis, no significant association between financing type and LTC costs remains. OLS regression for medical care costs shows a positive association with SHNHs that disappears in the IV analysis. After corrections for both observable and unobservable user characteristics, no statistically significant difference in total healthcare costs is found between SHNHs and to institutional provision. The large standard errors in the IV-analyses indicate that the statistical power may be insufficient to draw definitive conclusions based on these results. Changes in size and direction of the effect between OLS and IV may indicate that unobserved characteristics impact the outcome variable.

**Table 2:** OLS and IV analysis of location type and total healthcare costs

Healthcare costs in the year after moving to nursing home	Log long-term care costs (excluding housing costs)		Log costs of hospital and primary care (health insurance act)		Log total healthcare costs (excluding housing costs)	
	OLS	IV	OLS	IV	OLS	IV
Intercept	− 1.857^*^ (0.112)	− 1.782^*^ (0.170)	1.303^*^ (0.138)	1.619^*^ (0.299)	− 0.979^*^ (0.500)	− 1.002 (0.779)
Location type (0 = institutional; 1 = SHNH)	− 0.280^*^ (0.053)	2.448 (3.739)	1.749^*^ (0.059)	7.267 (4.564)	− 0.149^*^ (0.489)	2.051 (3.370)
Sex (0 = male; 1 = female)	0.077^*^ (0.021)	0.050 (0.044)	− 0.071^*^ (0.023)	− 0.128^*^ (0.054)	0.053^*^ (0.019)	0.031 (0.039)
Age (year move to NH) in years	0.004^*^ (0.001)	0.004^*^ (0.002)	− 0.010^*^ (0.001)	− 0.011^*^ (0.002)	− 0.002 (0.001)	− 0.002 (0.001)
Income (year move to NH) in €	0.001^*^ (0.001)	0.001 (0.001)	0.001 (0.001)	0.001 (0.001)	0.001^*^ (0.001)	0.001 (0.001)
Length of stay in the year after admission (days)	0.025^*^ (0.001)	0.025^*^ (0.001)	0.005^*^ (0.001)	0.005^*^ (0.001)	0.024^*^ (0.001)	0.024^*^ (0.001)
Case severity (ZZP) 5 (year move to NH, ref = ZZP 4)	− 0.127^*^ (0.030)	− 0.121^*^ (0.031)	− 1.931^*^ (0.033)	− 2.016^*^ (0.079)	0.065^*^ (0.027)	0.060^*^ (0.027)
Case severity (ZZP) 6 (year move to NH, ref = ZZP 4)	− 0.149^*^ (0.034)	− 0.084 (0.096)	− 1.043^*^ (0.037)	− 0.984^*^ (0.063)	0.070^*^ (0.031)	0.115 (0.075)
Case severity (ZZP) 7 (year move to NH, ref = ZZP 4)	− 0.214^*^ (0.051)	− 0.145 (0.111)	− 1.960^*^ (0.058)	− 1.944^*^ (0.062)	− 0.034 (0.047)	0.014 (0.090)
Case severity (ZZP) 8 (year move to NH, ref = ZZP 4)	0.091 (0.090)	0.167 (0.145)	− 1.252^*^ (0.100)	− 1.170^*^ (0.132)	0.219^*^ (0.083)	0.275^*^ (0.122)
Costs in year 2016 (0 = no; 1 = yes)	− 0.008 (0.022)	0.001 (0.025)	− 0.192^*^ (0.065)	− 0,257^*^ (0.076)	− 0.594 (0.491)	− 0.506 (0.787)
Costs in year 2017 (0 = no; 1 = yes)	1.369^*^ (0.025)	1.294^*^ (0.107)	2.799^*^ (0.031)	2,716^*^ (0.075)	1.815^*^ (0.026)	1.767^*^ (0.082)
Costs in year 2018 (0 = no; 1 = yes)	2.200^*^ (0.026)	2.168^*^ (0.056)	3.339^*^ (0.025)	3.134^*^ (0.172)	2.388^*^ (0.025)	2.368^*^ (0.048)
Postestimation tests						
Weak instruments (F-statistic)		19.787^*^		17.950^*^		19.101^*^
Wu-Hausman		0.547		1.575		0.436

*OLS* ordinary least squares regression, *IV* instrumental variable analysis, *NH* nursing home, *SHNH* Small-scale homelike nursing home

Values marked with * are significant at *p* < 0.05

The association between financing type and length of stay is shown in Table 3. OLS regression for length of stay in the two years after admission of stay yields a negative correlation with financing type, implying shorter length of stay in SHNHs. However, similar to the analyses on healthcare costs, the significance disappears after adjusting for selection effects. Similar to the analysis in Table 2, large standard errors in the IV-analyses point to a lack of power, which prevents drawing definitive conclusions.

**Table 3:** OLS and IV analysis of location type and length of stay

Length of stay	Length of stay in the year after admission (days)		Length of stay in the two years after admission (days)	
	OLS	IV	OLS	IV
Intercept	276.124^*^ (10.444)	276.714^*^ (12.853)	585.927^*^ (24.559)	585.070^*^ (30.269)
Location type (0 = institutional; 1 = SHNH)	− 6.698^*^ (1.914)	− 67.514^*^ (132.176)	− 11.759^*^ (4.500)	76.542 (312.897)
Sex (0 = male; 1 = female)	21.739^*^ (1.914)	22.370^*^ (1.832)	60.630^*^ (1.777)	59.714^*^ (3.996)
Age (year move to NH)	− 1.254^*^ (0.041)	− 1.240^*^ (0.052)	− 4.313^*^ (0.097)	− 4.332^*^ (0.121)
Income (year move to NH) in €	0.001^*^ (0.001)	0.001^*^ (0.001)	0.002^*^ (0.001)	0.002^*^ (0.001)
Case severity (ZZP) 5 (year move to NH, ref = ZZP 4)	− 14.895^*^ (1.068)	− 14.627^*^ (1.144)	− 53.325^*^ (2.510)	− 53.714^*^ (2.815)
Case severity (ZZP) 6 (year move to NH, ref = ZZP 4)	− 27.198^*^ (1.201)	− 28.287^*^ (2.632)	− 78.393^*^ (2.824)	− 76.813^*^ (6.262)
Case severity (ZZP) 7 (year move to NH, ref = ZZP 4)	− 7.870^*^ (1.870)	− 9.021^*^ (3.010)	− 47.284^*^ (4.396)	− 45.613^*^ (7.182)
Case severity (ZZP) 8 (year move to NH, ref = ZZP 4)	− 16.513^*^ (3.094)	− 17.716^*^ (4.065)	− 58.896^*^ (7.276)	− 57.150^*^ (9.680)
Costs in year 2016 (0 = no; 1 = yes)	− 9.100 (9.820)	− 12.571 (14.504)	− 33.659 (23.092)	− 28.619 (34.130)
Costs in year 2017 (0 = no; 1 = yes)	45.327^*^ (1.165)	46.306^*^ (2.451)	76.322^*^ (2.740)	74.900^*^ (5.595)
Costs in year 2018 (0 = no; 1 = yes)	87.904^*^ (0.842)	88.472^*^ (1.623)	253.611^*^ (1.980)	252.785^*^ (3.654)
Postestimation tests				
Weak instruments (F-statistic)		28.235^*^		28.230^*^
Wu-Hausman		0.214		0.080

*OLS* ordinary least squares regression, *IV* instrumental variable analysis, *NH* nursing home, *SHNH* Small-scale homelike nursing home

Values marked with * are significant at *p* < 0.05

### Sensitivity analyses

Sensitivity checks rendered similar results to the primary analyses (Supplementary material 3). We did not observe significant differences in facilities that offer both institutional care and SHNHs and facilities that offer one provision option.

### Qualitative results

Twenty stakeholder interviews were performed and analysed to enhance our understanding behind the cost drivers of SHNHs. Multiple respondents corroborated selection-effects in SHNHs. SHNHs typically target users with higher socioeconomic status. Moreover, SHNHs are considered an addition to the current LTC provider landscape, rather than a substitute for institutionalized care (i.e., in-kind financed nursing homes). Qualitative results suggest that SHNHs can tailor small-scale settings to specific needs and preferences, whereas institutional nursing homes typically provide care to a wider range of users on a larger scale. Access to institutional care is relatively limited in the Netherlands, which may minimize nursing home options, especially for higher case severity clients [32]. Concerns were raised that lower-income groups have restricted access to SHNHs.

*“Aging in place is an option to manage the increasing number of persons requiring long-term care. The more people can live at home*,* the better we can manage it. Small-scale nursing homes are an alternative to aging in place. People live there independently. They rent or buy an apartment and receive long-term care.”* (Health insurer/policymaker)

Furthermore, economic disadvantages in scale were mentioned by several respondents. Revenue per user to cover the running costs for SHNHs is relatively high, compared to institutional nursing homes. Night shifts are particularly difficult to organize cost-effectively, due to high personnel costs and relatively few LTC users. Respondents argue that a separation between (public) healthcare and (private) housing financing – opposed to institutional care, which comprises both elements within the same funding scheme – is required for viable exploitation of SHNHs because private funding for housing and additional services can be adjusted upwards to compensate for the losses to organize small-scale.

*“We focused on expanding*,* but to manage one or two sites is different to managing five*,* six*,* or seven. Other mechanisms for control are required. Small-scale homelike environments are vulnerable because of low staffing and low capacity. One or two people manage multiple sites*,* which may result in a loss of control.”* (Representative of an SHNH)

## Discussion

### Principal findings

In this study, we compared the total healthcare costs of institutional LTC and SHNHs. We found that unadjusted total healthcare costs are lower in SHNHs. However, IV analysis with adjustments for observable user characteristics (age, sex, income, case severity, length of stay) and unobservable user characteristics (self-selection) finds no difference in total healthcare costs. The IV analysis suffers from a lack of statistical power, which limits the conclusiveness of these results. However, the differences in effect size and direction between OLS and IV may indicate that unobserved characteristics have an effect on the outcome. The OLS may be biased by self-selection effects (unobserved characteristics), whereas our IV-analysis may be underpowered. These results demonstrate that SHNHs may potentially not lead to lower total healthcare costs if users are randomly assigned to either institutional LTC or SHNHs. Cost-shifting and selection effects may explain these findings. The qualitative research supports the findings that self-selection occurs.

An increase in home care use and SHNHs may lead to two types of cost-shifting. The first is a shift from LTC spending towards medical-care spending (under the HIA in the Netherlands). The medical care sector, particularly primary care and hospital care will likely experience an increased burden if the trend towards deinstitutionalization persists, since SHNHs do not offer medical care within their care package. Second, although we could not measure this quantitatively, our qualitative findings highlight that care within SHNHs may shift costs to private spending. SHNHs do not benefit from economies of scale. Viable exploitation of such organizations may only be possible if housing is arranged privately – which circumvents the financing ceiling on the compensation for housing as imposed by the in-kind package – and if additional services are commissioned by users. If, according to our findings, the public costs are higher for SHNHs compared to institutionalized homes, total public and private LTC costs may be even higher.

This study adds to the academic literature by focusing on SHNHs and comparing the costs of those settings to institutionalized homes. Our findings are partly supported by a similar study that used an IV approach [17]. Bakx et al. [17] find similar healthcare costs between home care and nursing home care prior to the healthcare reform. Three studies that do not use an IV approach find contrary results [18–20]. Kok et al. [18] analysed survey data before the LTC reform, finding lower costs in deinstitutionalized settings. Portrait and Koolman [19] only include long-term care costs including housing costs, finding that from the perspective of the LTC sector, deinstitutionalized care cost less. A study issued by the government focused on older persons that have experienced an impactful event (e.g., hip fracture), finding lower costs in this subgroup in deinstitutionalized settings. These results demonstrate the importance of incorporating cost shifting as well as correcting for patient selection.

### Strengths and limitations

Our study has two main strengths. First, our study uses national claim data and, therefore, has national coverage. Second, our focus on total healthcare costs, which include both long-term and medical care, allows for analysis of spillover effects. Potential spillovers to the medical sector may be an important factor in the rise of SHNHs [6]. Our results show the importance to adjust for selection, suggesting an IV approach using differential distance to isolate the effect of shifts in institutional LTC settings. The results may warn other health systems that consider such a shift that while the reform may appear to be cost saving, incorporating spillover effects and adjusting for selection effects may counterbalance any cost savings.

Several limitations apply to this study. First, the IV-analysis suffers from a lack of statistical power, due to the relatively small group of users in SHNHs. We do, however, argue that the IV-analysis holds value in this case, as it demonstrates that the OLS may be biased by unobserved characteristics. Further research may be able to include more recent data, which could improve statistical power. Second, a lack of information exists on private spending on housing and additional services. As we cannot quantitatively compare housing costs for SHNHs with those in institutional nursing homes, caution is warranted when interpreting whether SHNHs contribute to a sustainable LTC system or affects accessibility to LTC. Our qualitive data suggests that such costs may be higher in SHNHs, suggesting that total public and private cost of SHNHs may be higher than total costs for institutional provision. Third, medical-care costs were not differentiated within years, which may result in overestimation of medical-care use when persons move to a nursing home. To mitigate this limitation, we analysed healthcare costs in the year after moving to a nursing home – which eliminates costs in the run-up towards nursing home migration – and adjusted for the length of stay. Fourth, our results were not adjusted for ownership status (i.e., for-profit or not for-profit). However, additional analysis excluding organizations that offer both institutional and deinstitutionalized LTC – which often are not-for profit – suggests that for-profit provision may result in higher costs. Fifth, municipal address registration may be subject to errors. Inhabitants of the Netherlands register migration between addresses at their current municipality, but this registration is not always accurate. This may result in clusters of persons that, in reality, are not resident in an LTC setting. Our sensitivity analysis, however, showed that alternative definitions result in similar outcomes. Sixth, regional selection effects may occur as SHNHs are inclined to locate in affluent regions. We mitigated this limitation through the inclusion of income as a control variable, but some selection may remain. Seventh, mortality rates across groups may differ due to case severity differences, which can cause censoring of the survival outcome [33]. Although case severity is likely lower in SHNHs as compared to institutional nursing homes, these groups are not mutually exclusive and users may switch between groups, which may limit bias.

### Policy and research implications

The Netherlands has a high-cost, strongly institutionalized LTC system among OECD member states [4, 5]. While quality of care is generally regarded as high, upholding universal accessibility and quality is financially challenging. The shift towards SHNHs may generate public savings in terms of housing arrangements, but savings on total healthcare costs can be subject to overestimation due to user-selection effects and spillover effects to the medical care sector.

Access to SHNHs offers both benefits and drawbacks: a diverse set of LTC providers offers a wide range of options for an increasingly diverse older population. SHNHs are thus potentially able to provide more patient-centred care. An increase in private payments for SHNHs may be accompanied with (a perception of) higher quality of care. However, concerns are raised whether access to SHNHs is guaranteed for lower-income groups, as private contributions are relatively high. Moreover, the principle of solidarity may be undermined when high-income groups shift towards SHNHs and contribute less through co-payments. Furthermore, self-selection of low-cost users towards SHNHs may increase the care need and severity of the LTC users - and thereby average costs - of institutional LTC. In that sense, the financial viability of private LTC providers is a concern in the longer term [34]. The market may suffer from volatility, which can endanger access.

This study adds to existing knowledge on SHNHs using an instrumental variable to correct for observable and unobservable characteristics. Future research is necessary to assess the full implications of SHNHs on total costs, including private payments. Insight into both public and private spending may provide policymakers with guidance in trade-offs on access and solidarity. Furthermore, research into quality of care in SHNHs may be essential for assessing the added value.

## Conclusion

SHNHs have been stimulated by the Dutch government with the aim to contain healthcare costs. However, we do not find a reduction in total healthcare costs for SHNHs, correcting for spillover costs, confounders, and selection effects. Furthermore, beyond the cost-containment argumentation, pursuing deinstitutionalization of LTC can have negative implications for equity and solidarity.

## Supplementary Information

Supplementary Material 1.

Supplementary Material 2.

Supplementary Material 3.

## Data Availability

The data that support the findings of this study are available from Statistics Netherlands but restrictions apply to the availability of these data, which were used under license for the current study, and so are not publicly available. Data are however available from the authors upon reasonable request and with permission of Statistics Netherlands. For further information, contact microdata@cbs.nl.

## Abbreviations

CBS: Centraal Bureau voor de Statistiek [Statistics Netherlands]
CIZ: Centrum Indicatiestelling Zorg [Care assessment centre]
GDP: Gross Domestic Product
GP: General Practitioner
HCP: Home Care Package
HIA: Health Insurance Act
IV: Instrumental Variable(s)
LTC: Long-Term Care
MCP: Modular Care Package
NH: Nursing Home
NZa: Nederlandse Zorgautoriteit [Dutch healthcare authority
OECD: Organisation for Economic Co-operation and Development
OLS: Ordinary Least Squares
PB: Personal Budget
SHNH: Small-scale homelike nursing home
