# Supplementary material for: Total healthcare costs of deinstitutionalized long-term care provision in the Netherlands: an instrumental variable analysis
Source: BMC Health Serv Res. 2025 Apr 10;25:529. doi: 10.1186/s12913-025-12693-x (PMC11984009; doi:10.1186/s12913-025-12693-x)
Supplement: Supplementary file 1 — Supplementary Material 1. [file 12913_2025_12693_MOESM1_ESM.docx]

Total healthcare costs of deinstitutionalized long-term care provision in the Netherlands: an instrumental variable analysis

**Author information**

1.Erik M.E. Wackers (Corresponding author)^1^

Email: Erik.Wackers@radboudumc.nl

2. Florien M. Kruse^1,2^

3.Bart (H.) J.J.M. Berden^1^

4.Simone A. van Dulmen^1^

5.Niek W. Stadhouders^1^

6.Patrick P.T. Jeurissen^1,2^

**Affiliations**

^1^ Radboud University Medical Center, Radboud Institute for Health Sciences, IQ healthcare, Nijmegen, the Netherlands

^2^ Ministry of Health, Welfare, and Sport, The Hague, the Netherlands

**Supplementary material 1.** Tariffs in long-term care in the Netherlands

**Table A1.** Case severity and tariffs for institutional long-term care (LTC), home care package (HCP) and personal budget (PB)*. Values are € per day (Year 2017) .

|  | Institutional LTC (without treatment) | HCP | PB |
| --- | --- | --- | --- |
| **ZZP VV 4** Assisted living with intensive support and extensive nursing | 118,71 | 116,8 | 96,66 |
| **ZZP VV 5** Nursing home care with extensive dementia care | 162,92 | 161,17 | 131,15 |
| **ZZP VV 6** Nursing home care with extensive personal care and nursing | 163,23 | 161,15 | 131,15 |
| **ZZP VV 7** Nursing home care with intensive care, with focus on supervision (often behavioural problems) | 191,76 | 189,19 | 164,28 |
| **ZZP VV 8** Nursing home care with intensive care, with focus on personal care/ nursing (problems with activities of daily living and cognitive) | 223,77 | 219,81 | 196,06 |

Source: NZa Prestatiebeschrijvingen en tarieven zorgzwaartepakketten – BR/REG-17137b en BR/REG-17137f

*Modular care packages (MCP) are not included in this table since the composition of modules varies per user which results in costs variation. Tariffs per module for 2017 can be found at: https://puc.overheid.nl/nza/doc/PUC_21608_22/
