# Supplementary material for: Total healthcare costs of deinstitutionalized long-term care provision in the Netherlands: an instrumental variable analysis
Source: BMC Health Serv Res. 2025 Apr 10;25:529. doi: 10.1186/s12913-025-12693-x (PMC11984009; doi:10.1186/s12913-025-12693-x)
Supplement: Supplementary file 2 — Supplementary Material 2. [file 12913_2025_12693_MOESM2_ESM.docx]

Total healthcare costs of deinstitutionalized long-term care provision in the Netherlands: an instrumental variable analysis

**Author information**

1.Erik M.E. Wackers (Corresponding author)^1^

Email: Erik.Wackers@radboudumc.nl

2. Florien M. Kruse^1,2^

3.Bart (H.) J.J.M. Berden^1^

4.Simone A. van Dulmen^1^

5.Niek W. Stadhouders^1^

6.Patrick P.T. Jeurissen^1,2^

**Affiliations**

^1^ Radboud University Medical Center, Radboud Institute for Health Sciences, IQ healthcare, Nijmegen, the Netherlands

^2^ Ministry of Health, Welfare, and Sport, The Hague, the Netherlands

**Supplementary material 2**. Topic guide

**Table A2**. Topic guide semi structured interviews

| **Developments** | How would you describe the developments regarding (small-scale) deinstitutionalized care in the Netherlands? |
| --- | --- |
| **Opportunities and barriers - financial** | What are the opportunities for (small-scale) deinstitutionalized providers to contribute to affordable healthcare according to you? |
|  | What are the barriers for (small-scale) deinstitutionalized providers to contribute to affordable healthcare according to you? |
| **Opportunities and barriers - quality** | What are the opportunities for (small-scale) deinstitutionalized providers to contribute to quality of care according to you? |
|  | What are the barriers for (small-scale) deinstitutionalized providers to contribute to quality of care according to you? |
| **Opportunities and barriers - access** | What are the opportunities for (small-scale) deinstitutionalized providers to contribute to access of care for long-term care needs? |
|  | What are the barrier for (small-scale) deinstitutionalized providers to contribute to access of care for long-term care needs? |
