# Supplementary material for: Total healthcare costs of deinstitutionalized long-term care provision in the Netherlands: an instrumental variable analysis
Source: BMC Health Serv Res. 2025 Apr 10;25:529. doi: 10.1186/s12913-025-12693-x (PMC11984009; doi:10.1186/s12913-025-12693-x)
Supplement: Supplementary file 3 — Supplementary Material 3. [file 12913_2025_12693_MOESM3_ESM.docx]

Total healthcare costs of deinstitutionalized long-term care provision in the Netherlands: an instrumental variable analysis

**Author information**

1.Erik M.E. Wackers (Corresponding author)^1^

Email: Erik.Wackers@radboudumc.nl

2. Florien M. Kruse^1,2^

3.Bart (H.) J.J.M. Berden^1^

4.Simone A. van Dulmen^1^

5.Niek W. Stadhouders^1^

6.Patrick P.T. Jeurissen^1,2^

**Affiliations**

^1^ Radboud University Medical Center, Radboud Institute for Health Sciences, IQ healthcare, Nijmegen, the Netherlands

^2^ Ministry of Health, Welfare, and Sport, The Hague, the Netherlands

**Supplementary material 3.** Sensitivity analyses

**Table A3.1.** Higher minimal thresholds for clusters of users (e.g. ten or fifteen individuals) could result in an underestimation of locations. These identifying conditions for deinstitutionalized homes were relaxed or restricted.

**Table A3.2.** Ordinary least squares (OLS) regression using differential distance as predictor for location type (N=68,168). Differential distance = distance to closest deinstitutionalized provider – distance to closest provider.

|  | User choice for institutional or deinstitutionalized NH |
| --- | --- |
|  | OLS |
| Differential distance | -0,002* |
| Sex (0=male; 1=female) | 0,011* |
| Age (year move to NH) | 0,001* |
| Income (year move to NH) in € | 0,001* |
| Length of stay in the year after admission (days) | -0,001* |
| Case severity (ZZP) 5 (year move to NH, ref=ZZP 4) | 0,004* |
| Case severity (ZZP) 6 (year move to NH, ref=ZZP 4) | -0,018* |
| Case severity (ZZP) 7 (year move to NH, ref=ZZP 4) | -0,019* |
| Case severity (ZZP) 8 (year move to NH, ref=ZZP 4) | -0,020* |
| Costs in year 2016 (0=no; 1=yes) | -0,058* |
| Costs in year 2017 (0=no; 1=yes) | 0,017* |
| Costs in year 2018 (0=no; 1=yes) | 0,012* |

Values marked with * are significant at p < 0.05.

**Table A3.3.** Regression models (OLS and IV) for long-term care costs in the year after move to nursing home, without correction for case severity (n=75.146).

|  | Long-term care costs | |
| --- | --- | --- |
|  | OLS | IV |
| Location type (0=institutional; 1=deinstitutionalized) | -0.218* | 4,160 |
| Sex (0=male; 1=female) | 0.077* | 0.032 |
| Age (year move to NH) | -0.003* | -0.004* |
| Income (year move to NH) in € | 0,001* | 0,001 |
| Length of stay in the year after admission (days) | 0.025* | 0.025* |
| Costs in year 2016 (0=no; 1=yes) | -0.020 | -0,001 |
| Costs in year 2017 (0=no; 1=yes) | 1,394* | 1,275* |
| Costs in year 2018 (0=no; 1=yes) | 2,138* | 2,086* |

(IV = Instrumental variables; OLS = Ordinary Least Squares). Values marked with * are significant at p < 0.05.

**Table A3.4.** Regression models (OLS and IV) for total healthcare costs in the year after move to nursing home; corrections for age, sex, and costs in years 2016-2018 (n=75.149).

|  | Total healthcare costs | |
| --- | --- | --- |
|  | OLS | IV |
| Location type (0=institutional; 1=deinstitutionalized) | -0,164* | 3,603 |
| Sex (0=male; 1=female) | 0,236* | 0,220* |
| Age (year move to NH) | -0,016* | -0,018* |
| Costs in year 2016 (0=no; 1=yes) | 0,691 | 0,824 |
| Costs in year 2017 (0=no; 1=yes) | 2,919* | 2,833* |
| Costs in year 2018 (0=no; 1=yes) | 5,460* | 5,424* |

(IV = Instrumental variables; OLS = Ordinary Least Squares). Values marked with * are significant at p < 0.05.

|  |
| --- |

**Table A3.5.** Regression models (OLS and IV) for total healthcare costs in the year after move to nursing home, correction for locations that offer both institutional and deinstitutionalized long-term care (n=75.141).

|  | Total healthcare costs | |
| --- | --- | --- |
|  | OLS | IV |
| Location type (0=institutional; 1=deinstitutionalized) | -0,144* | 0,498 |
| Sex (0=male; 1=female) | 0,053* | 0,047 |
| Age (year move to NH) | -0,002 | -0,002 |
| Income (year move to NH) in € | 0,001* | 0,001 |
| Length of stay in the year after admission (days) | 0,024* | 0,024* |
| Case severity (ZZP) 5 (year move to NH, ref=ZZP 4) | 0,067* | 0,069* |
| Case severity (ZZP) 6 (year move to NH, ref=ZZP 4) | 0,073* | 0,088 |
| Case severity (ZZP) 7 (year move to NH, ref=ZZP 4) | -0,031 | -0,013 |
| Case severity (ZZP) 8 (year move to NH, ref=ZZP 4) | 0,223* | 0,243* |
| Costs in year 2016 (0=no; 1=yes) | -0,593 | -0,566 |
| Costs in year 2017 (0=no; 1=yes) | 1,815* | 1,801* |
| Costs in year 2018 (0=no; 1=yes) | 2,388* | 2,382* |
| Location offers both institutionalized and deinstitutionalized long-term care (0=no; 1=yes) | 0,022 | 0,046 |

(IV = Instrumental variables; OLS = Ordinary Least Squares). Values marked with * are significant at p < 0.05.
